# Supplementary material for: Cross-Species Transmission of Rabbit Hepatitis E Virus to Pigs and Evaluation of the Protection of a Virus-like Particle Vaccine against Rabbit Hepatitis E Virus Infection in Pigs
Source: Vaccines (Basel). 2022 Jun 30;10(7):1053. doi: 10.3390/vaccines10071053 (PMC9320745; doi:10.3390/vaccines10071053)
Supplement: Supplementary file 1 [file vaccines-10-01053-s001.zip › vaccines-1766179-supplementary.pdf]

Supplementary Materials:

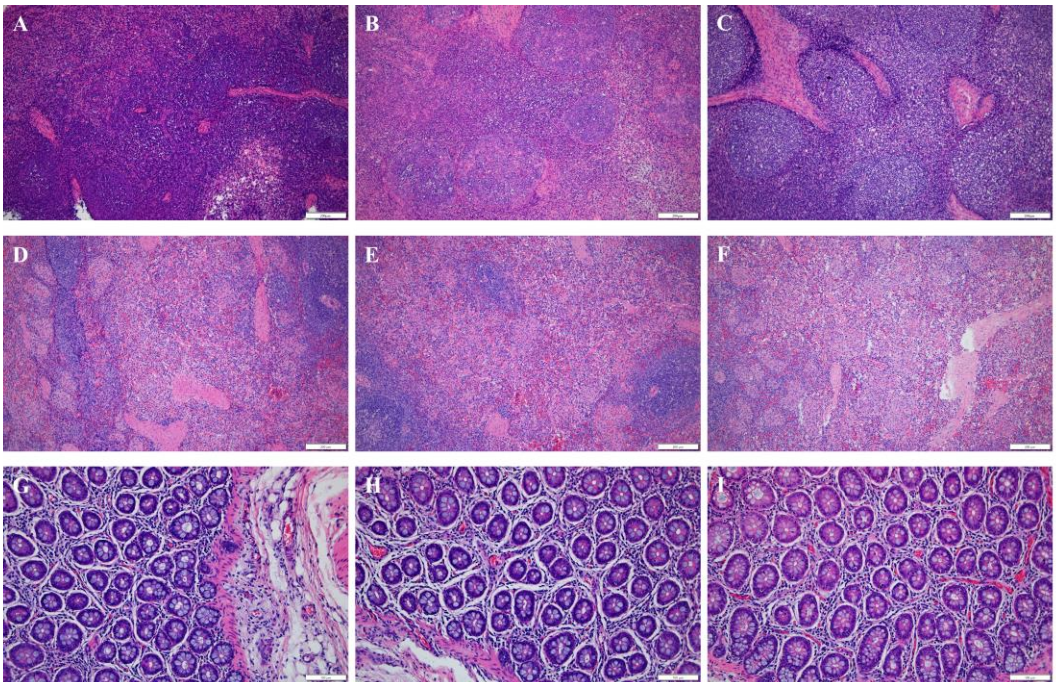

Figure S1. Histopathological lesions in abdominal organs: **(A)** to **(C)** mesenteric lymph node; **(D)** to **(F)** spleen; and **(G)** to **(I)** large intestine; **(A)**, **(D)**, and **(G)** show tissues from pigs in the negative control; **(B)**, **(E)**, and **(H)** show tissues from pigs in the positive control; **(C)**, **(F)**, and **(I)** show tissues form pigs in the vaccinated group.

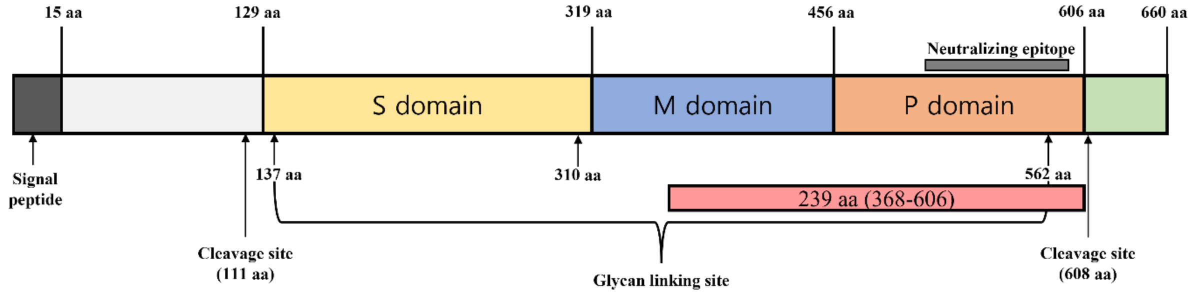

Figure S2. Schematic diagram of HEV for VLP vaccine

Table S1. Determination of IL-12 levels in pigs

week	Negative control			Positive control				Vaccinated				
	1	2	3	4	5	6	7	8	9	10	11	12
0	0.90	0.95	1.00	1.22	0.96	1.02	0.98	0.84	0.93	1.25	1.24	1.17
2	0.75	0.83	0.84	0.91	1.16	0.97	0.81	1.25	0.79	0.91	0.95	0.87
4	0.75	0.78	1.11	0.80	1.22	1.01	1.04	3.01	2.20	2.37	2.99	3.78
6	0.85	1.13	0.94	0.83	0.91	1.20	0.84	1.01	1.14	0.99	1.21	1.13
8	0.94	1.13	0.73	0.83	0.77	0.95	1.05	1.28	1.19	0.85	0.83	1.27
10	1.13	1.16	0.74	1.22	0.88	0.81	1.23	0.74	1.19	1.15	0.74	1.18

Table S2. Determination of IFN-γ levels in pigs

week	Negative control			Positive control				Vaccinated				
	1	2	3	4	5	6	7	8	9	10	11	12
0	22.74	23.98	30.47	34.43	39.06	23.44	24.64	32.90	22.73	33.94	22.35	37.92
2	34.56	31.66	25.38	31.10	36.00	37.92	33.31	34.74	26.05	38.69	35.12	24.28
4	29.09	36.24	25.03	40.45	24.49	25.78	40.71	142.59	152.08	145.83	136.83	156.72
6	25.78	27.25	21.85	26.61	31.95	38.82	32.38	90.77	126.68	109.84	125.86	129.90
8	34.65	25.75	38.01	37.87	29.14	27.37	35.71	20.59	40.13	22.37	38.47	39.84
10	37.82	37.22	35.68	40.62	38.58	20.67	38.62	34.51	29.99	28.27	20.45	22.17
